# The Association Between Sensory Impairment and Adherence to COVID-19 Prevention Measures in the Adult California Health Interview Survey Population

**DOI:** 10.3390/vision9020040

**Published:** 2025-05-02

**Authors:** Catherine T. Cascavita, Ahmad Santina, Ken Kitayama, Fei Yu, Victoria L. Tseng, Anne L. Coleman

**Affiliations:** 1 David Geffen School of Medicine, University of California, Los Angeles, CA 90095, USAvtseng@mednet.ucla.edu (V.L.T.); 2 Department of Ophthalmology, Stein and Doheny Eye, Los Angeles, CA 90095, USAkkitayama@mednet.ucla.edu (K.K.); fyu@ucla.edu (F.Y.); 3 Department of Biostatistics, Fielding School of Public Health, University of California, Los Angeles, CA 90095, USA; 4 Department of Epidemiology, Fielding School of Public Health, University of California, Los Angeles, CA 90095, USA

**Keywords:** sensory impairment, COVID-19, public policy, mitigation strategies

## Abstract

This study explores the association between vision/hearing impairment and COVID-19 prevention strategies in the 2020 and 2021 California Health Interview Survey (CHIS). This cross-sectional study used data from the 2020 and 2021 CHIS. The exposure of interest was self-reported history of sensory impairment. The outcome of interest was adherence to COVID-19 mitigation strategies defined as obtaining a COVID-19 vaccine, face mask adherence, hand washing, social distancing, and not gathering with non-household members. Logistic regression models examined the association between sensory impairment and adherence to COVID-19 mitigation strategies, controlling for age, sex, race and ethnicity, general health status, and household income. All analyses were weighted according to the CHIS sampling design. With 24,453 California adults representing 29,649,837 people, the weighted prevalence of sensory impairment was 6.1% (1,808,640/29,649,837). The regression revealed that adults with sensory impairment were 80% more likely not to maintain social distancing (odds ratio: 1.80, 95%CI: 1.03–3.13, *p* = 0.04) compared to those without impairment. No significant differences were found for adherence to other COVID-19 strategies. Individuals with sensory impairment may have increased difficulty with physical distancing due to their underlying impairment. Further studies are needed to explore risk reduction strategies for COVID-19 and the transmission of other infections for those with sensory impairment.

## 1. Introduction

The World Health Organization (WHO) declared the coronavirus disease 2019 (COVID-19) a global pandemic in March 2020, resulting in a significant healthcare and public health burden [1,2]. Various preventive measures were implemented to limit the spread of COVID-19, including hand hygiene, isolation, personal protective equipment, and social distancing [3]. Prior studies hypothesized that prevention measures such as social distancing and the wearing of standard face masks may be difficult to follow, especially for those with sensory impairment [4,5]. These difficulties may stem from disturbances in sound, visual cues, and reduced visual fields, disproportionately affecting individuals with sensory impairment. Face masks muffle sound [6], block facial expressions [7], and restrict the ability to lip read [8], significantly impacting those with hearing impairments. Also, face masks may obstruct the lower visual fields, impair gait and balance, and further increase fall risk in those with visual impairment [9,10]. While COVID-19 prevention measures may be difficult to follow, adherence to these measures during the pandemic in individuals with reported sensory impairment is not well understood. This is of particular concern, given that vulnerable groups such as those with disabilities have been disproportionately affected by COVID-19, leading to greater health inequities [11]. For example, in an observation study of 38 participants, Epstein et al. (2021) found that COVID-19 exacerbated common barriers faced by individuals with disabilities, such as access to healthcare, resources, and employment opportunities [12].

In May 2023, the WHO declared that COVID-19 was no longer a public health emergency or global pandemic [13]. Yet, multiple negative consequences of the COVID-19 pandemic persisted, such as worse health inequities and increased anxiety, stress, and depression [14,15,16]. Thus, there remains a need for an investigation into the effectiveness of public health measures during the COVID-19 pandemic to influence future pandemic preparedness strategies. This is especially important given that emerging infectious diseases are projected to continue increasing with time, likely resulting in another pandemic [17]. Moreover, the population in the United States is aging, resulting in a continued increase of sensory impairments from age-related conditions in the coming years [18,19,20]. These factors suggest that a future pandemic would exacerbate health inequities to a larger magnitude than the COVID-19 pandemic without further preparedness and intervention.

This study examines the relationship between sensory impairments (vision and/or hearing) and adherence to COVID-19 mitigation strategies in order to provide generalizable insights for future public health interventions for individuals with sensory impairment. Specifically, it examined associations between vision and hearing impairment and the wearing of face masks, handwashing, COVID-19 vaccination, social distancing, and non-household member gatherings in the 2020–2021 California Health Interview Survey (CHIS).

## 2. Methods

A cross-sectional study from the 2020 and 2021 California Health Interview Survey (CHIS) was conducted. The CHIS is the nation’s largest state health survey and utilizes sampling strata representing the entire state of California, including all 58 counties. Survey responses were obtained through mixed-mode surveys using telephone calls and online surveys in six languages: English, Spanish, Chinese, Korean, Vietnamese, and Tagalog. The CHIS collects information on health status, conditions, mental health, health behaviors, and living environment, among many more. The Institutional Review Board (IRB) of the University of California, Los Angeles, approved this study.

All adult CHIS participants (greater than 18 years of age) were included in this study. The exposure of interest was having sensory impairment, defined as answering “Yes” to the question, “are you blind or deaf, or do you have a severe vision or hearing problem?” on the CHIS. The outcome of interest was self-reported adherence to COVID-19 mitigation strategies, which included the wearing of face masks, maintaining social distancing (greater than 6 feet apart), hand washing, and obtaining a vaccine (Table S1). Each mitigation measure was assessed via questionnaire and defined as a separate outcome for this study. All mitigation measures in CHIS were included.

Demographic information included sex, age, race/ethnicity, and self-reported health status. Descriptive statistics were used to describe the study population at the time of survey. Categorical variables were reported as percentage (%) with accompanying 95% confidence intervals (CI). Chi-squared tests were used to perform univariable comparisons of categorical variables, while Mann U Whitney tests were used for continuous variables. Logistic regression models were used to examine the association between sensory impairment and adherence to each COVID-19 mitigation measure, adjusting for age, sex, race/ethnicity, self-reported general health status, and annual household income. All analyses were weighted according to the CHIS sampling design. A *p*-value of <0.05 was considered statistically significant. Statistical analysis was conducted using SAS version 9.4.

## 3. Results

A total of 24,453 adult individuals were sampled in this study, which represented a weighted population size of 29,649,837 based on the CHIS sampling design (Table 1). The weighted prevalence of sensory impairment was 6.1% (1,808,640/29,649,837). There were similar proportions of males and females in those who reported sensory impairment compared to those who did not (*p* = 0.70). The largest proportion of individuals identified as White (N = 12,008,184, 40.5%) and Latino (N = 10,970,439, 37.0%). Individuals who reported sensory impairment were noticeably older in age; 17.5% of sensory-impaired individuals were 80 years or older compared to 3.8% of those without sensory impairment (*p* < 0.001). Additionally, a higher proportion of sensory-impaired individuals reported poor health compared to those without sensory impairment (12.6% vs. 2.3%, *p* < 0.001).

Table 2 provides univariable comparisons of COVID-19 mitigation measures in individuals with sensory impairment versus those without sensory impairment. There was no statistically significant difference in attitudes towards COVID-19 vaccination between groups (*p* = 0.59). A higher proportion of individuals with sensory impairment reported not leaving their home compared to individuals without sensory impairments (sensory impairment: 3.4% vs. no sensory impairment: 1.0%, *p* < 0.0001). Similarly, a larger proportion of individuals with sensory impairment reported that they did not gather with persons not living in a household compared to individuals without sensory impairment (sensory impairment: 46.3% vs. no sensory impairment: 39.3%, *p* = 0.0003).

Table 3 summarizes results from logistic regression analyses of the association between sensory impairment and adherence to COVID-19 prevention strategies. In unadjusted analyses, individuals with sensory impairment were 25% less likely to gather with non-household members (unadjusted odds ratio [OR] 0.75, 95% CI = 0.64–0.87, *p* < 0.0001). There was no longer a statistically significant association after adjusting for age, sex, race, ethnicity, self-reported health status, and annual household income (adjusted OR 1.06, 95% CI = 0.90–1.25, *p* = 0.48). Additionally, sensory impairment was not found to significantly increase the odds of nonadherence to social distancing in the unadjusted model (unadjusted OR 1.29, 95% CI = 0.72–2.29, *p* = 0.39). However, in fully adjusted analysis, individuals with sensory impairment were found to be 80% more likely never to maintain six feet distance from others (adjusted OR 1.80, 95% CI= 1.03–3.13, *p* = 0.04). There were no statistically significant differences in adherence to the remaining COVID-19 mitigation measures for individuals with versus without sensory impairment.

## 4. Discussion

The risk of future pandemics is expected to increase with time. Thus, it is imperative that we learn from the COVID-19 pandemic to better inform future health policy and public health measures, especially for vulnerable groups of individuals. Our study, assessing the association of sensory impairment and adherence to COVID-19 mitigation strategies, found that individuals with sensory impairment were more likely to not adhere to social distancing after adjusting for age, race/ethnicity, health status, and income compared to those without sensory impairment. There were no statistically significant associations between sensory impairment and adherence to other COVID-19 mitigation measures, including face mask use, hand hygiene, gathering with others, and receipt of the COVID-19 vaccine.

There are several potential explanations for our finding of decreased adherence to social distancing in individuals with sensory impairment. Individuals with sensory impairment may face challenges adhering to social distancing due to difficulty visually identifying an appropriate distance to maintain with others. In a comprehensive review, Feghali et al. (2024) found that individuals with sensory impairment often bump into others and maintain a closer distance than nonimpaired individuals [21]. Traditional mobility aids, such as white canes and guide dogs, help visually impaired individuals detect obstacles, curbs, and steps; however, these aids are not designed to assist users in maintaining a recommended physical distance possibly leading to inadequate social distancing. Various studies have reported on technological developments that may address the needs of individuals with sensory impairment, specifically those related to social distancing. For example, Shrestha et al. (2020) developed a smartphone application that detects crowds and estimates the distance from the user to the identified crowd. As the distance shortens, the application produces an audio alert. This alert helps the impaired individual maintain social distancing [22]. Additional studies are needed to examine the specific strategies that would be most beneficial to aid with social distancing for those with vision and hearing impairment and its subsequent risk reduction effect on viral and other infectious illnesses.

COVID-19 mitigation measures were essential in reducing the transmission of the virus and reducing hospital admissions, morbidity, and mortality. Although these measures were necessary and beneficial, they introduced challenges for people with sensory impairments, including communication and vision difficulties [23]. In an observational study of 163 participants, Bubbico et al. (2021) found that face masks caused the greatest discomfort among the mitigation measures due to the created communication barriers, especially for those with hearing impairments [24]. Hearing-impaired individuals often rely on lip reading and facial expressions to aid effective communication [25]. Face masks obstruct facial expressions and the ability to lip-read, which may explain the reported discomfort among individuals with hearing impairments, as they may lose valuable communication aids. Also, face masks have been reported to muffle sound and reduce speech intelligibility. Bottalico et al. (2020) found in a cohort study of 40 participants with normal hearing that cloth face masks attenuate sound even more than N95 and surgical masks [6]. Sound attenuation from face masks impacts all users, but individuals with hearing impairments may be disproportionately affected due to their underlying disabilities. Surprisingly, the present study found no differences in adherence to face masks in individuals with versus without sensory impairment. It is possible that individuals in California found greater protective value than discomfort in adhering to the wearing of face masks. Moreover, individuals may have relied on alternative face masks, such as clear shields or face masks with see-through windows, although using these alternative mask options was not specifically assessed in CHIS. Our adjusted model also found no difference in adherence to hand hygiene, non-household member gatherings, and wanting/receiving the COVID-19 vaccine. These findings may be a result of our exposure variable. Our study categorized vision and hearing impairment into one group, sensory impairment, which may have dampened observable adherence difficulties specific to hearing impairment or vision impairment. Further studies separately examining associations between vision and hearing impairment and likelihood of adherence to infection prevention and mitigation measures would be of benefit for public health considerations for vulnerable populations in a future pandemic.

This study has various limitations worth noting. This study may suffer from response bias, given that the inclusion of individuals into the dataset was contingent on completing a survey. Due to the possible stigma associated with non-adherence to mitigation COVID-19 measures during the pandemic, individuals may have been less inclined to respond truthfully. The CHIS survey did not provide respondents with clear guidance or definitions for the term “severe”, potentially leading to ambiguity in interpretation and inaccurate reporting of severity level. Also, given the survey data’s self-reported nature, it is impossible to stratify sensory impairment by category or severity, limiting sub-group analyses.

In summary, this study found an association between sensory impairment and non-adherence to social distancing, defined as maintaining six feet of social distance. Thus, physical distancing may be more difficult for those with sensory impairment. Sensory impaired individuals may benefit from alternative recommendations that limit the spread of infectious diseases.

## Figures and Tables

**Table 1:** Characteristics of 2020–2021 California Health Interview Survey study population.

	Total Population n = 24,453 N = 29,649,837		Sensory Impairment n = 1492 N = 1,808,640		No Sensory Impairment n = 22,961 N = 27,841,197		*p*-Value
	Percent (%)	95% CI * (%)	Percent (%)	95% CI * (%)	Percent (%)	95% CI * (%)	
**Age (years)**							
18–39	38.5	38.5–38.5	19.1	16.2–22.3	39.8	39.6–40.0	*p* < 0.0001
40–59	32.0	31.7–32.4	25.3	22.4–28.4	32.5	32.0–32.9	
60–79	24.8	24.5–25.2	38.1	34.6–41.7	24.0	23.6–24.4	
80+	4.6	4.5–4.8	17.6	15.3–20.1	3.8	3.6–4.0	
**Sex**							
Male	49.0	49.0–49.0	48.4	45.2–54.9	49.1	48.9–49.3	*p* = 0.70
Female	51.0	51.0–51.0	51.6	48.4–54.9	50.9	50.7–51.1	
**Race/ethnicity**							
White	40.5	40.5–40.5	39.0	35.7–42.4	40.6	40.3–40.8	*p* = 0.08
Latino	37.0	37.0–37.0	41.1	37.2–45.0	36.7	36.5–37.0	
Black	5.6	5.6–5.6	4.3	3.2–5.7	5.7	5.6–5.8	
American Indian	0.5	0.4–0.5	0.7	0.3–1.7	0.5	0.4–0.5	
Asian	13.8	13.8–13.8	13.1	10.7–16.1	13.9	13.7–14.1	
Other	2.6	2.6–2.7	1.8	1.2–2.9	2.7	2.6–2.8	
**General Health**							
Excellent	17.6	16.9–18.2	7.2	5.7–9.0	18.2	17.5–18.9	*p* < 0.0001
Very Good	34.1	33.3–34.9	19.5	17.0–22.2	35.0	34.2–35.8	
Good	31.9	31.1–32.8	33.4	30.2–36.7	31.8	30.9–32.7	
Fair	13.6	13.0–14.2	27.3	24.0–30.8	12.7	12.1–13.3	
Poor	2.9	2.6–3.2	12.6	9.8–16.2	2.3	2.0–2.6	

* 95% Confidence Interval (CI) derived using 80 jackknife replications. n = number of observations; N = weighted sample.

**Table 2:** Survey results for California adults completing 2020-2021 California Health Interview Survey.

	Total Population n = 24,453 N = 29,649,837		Sensory Impairment n = 1492 N = 1,808,640		No Sensory Impairment n = 22,961 N = 27,841,197		*p*-Value
	Percent (%)	95% CI * (%)	Percent (%)	95% CI * (%)	Percent (%)	95% CI * (%)	
**Would get COVID vaccine if available?**							
Yes	19.3	18.6–19.9	20.7	17.7–24.1	19.2	18.5–19.9	*p* = 0.59
No	23.8	23.1–24.5	23.2	20.4–26.3	23.8	23.1–24.6	
Received dose	57.0	56.1–57.8	56.1	52.6–59.5	57.0	56.2–57.8	
**How often wore face covering?**							
Never	3.5	3.2–3.9	3.7	2.6–5.1	3.5	3.2–3.9	*p* < 0.0001
Sometimes	10.4	9.9–10.9	9.1	7.3–11.5	10.4	9.9–11.0	
Usually	15.6	15.0–16.2	13.2	10.9–15.9	15.8	15.2–16.4	
Always	69.4	68.7–70.1	70.7	67.0–74.1	69.3	68.6–70.0	
Did not leave home	1.1	0.9–1.3	3.4	1.9–5.8	1.0	0.8–1.1	
**How often washed hands?**							
Never	2.4	2.2–2.7	2.4	1.6–3.7	2.5	2.2–2.7	*p* < 0.0001
Sometimes	8.8	8.3–9.3	9.4	7.2–12.2	8.8	8.3–9.3	
Usually	20.4	19.7–21.1	21.2	18.1–24.6	20.3	19.6–21.1	
Always	67.3	66.5–68.1	63.7	59.7–67.5	67.5	66.8–68.3	
Did not leave home	1.1	0.9–1.3	3.4	1.9–5.8	1.0	0.8–1.1	
**How often maintained 6ft distance?**							
Never	1.8	1.6–2.1	2.2	1.3–3.8	1.8	1.5–2.0	*p* < 0.0001
Sometimes	13	12.5–13.6	10.5	8.6–12.9	13.2	12.6–13.8	
Usually	39	38.2–39.8	36.6	33.4–39.9	39.1	38.3–40.0	
Always	45.1	44.1–46.0	47.3	43.3–51.4	44.9	44.0–45.9	
Did not leave home	1.1	0.9–1.3	3.4	1.9–5.8	1.0	0.8–1.1	
**Gathered with persons not living in household?**							
Yes	60.3	59.4–61.2	53.7	49.8–57.6	60.8	59.9–61.6	*p* = 0.0003
No	39.7	38.8–40.6	46.3	42.4–50.2	39.3	38.4–40.1	

* 95% Confidence Interval (CI) derived using 80 jackknife replications. n = number of observations; N = weighted sample.

**Table 3:** Unadjusted and adjusted odds ratios for association between sensory impairment and COVID-19 prevention strategy adherence.

Outcome (Reference: No Sensory Impairment)		OR	95% CI	*p*-Value
**Want or Received COVID-19 Vaccine**	Unadjusted	1.03	0.87–1.23	0.71
	Adjusted *	0.94	0.77–1.13	0.50
**Face Mask Non-adherence**	Unadjusted	1.07	0.73–1.56	0.73
	Adjusted *	1.08	0.75–1.55	0.66
**Hand Hygiene Non-adherence**	Unadjusted	1.00	0.63–1.61	0.98
	Adjusted *	0.94	0.59–1.50	0.79
**Social Distancing Non-Adherence**	Unadjusted	1.29	0.72–2.29	0.39
	Adjusted *	1.80	1.03–3.13	0.04
**Non-household Member Gatherings**	Unadjusted	0.75	0.64–0.87	0.00
	Adjusted *	1.06	0.90–1.25	0.48

OR: odds ratio; CI: confidence interval. * Adjusted for age, sex, race/ethnicity, self-reported health status, and annual household income.

## Data Availability

The original data presented in the study are openly available from the UCLA Center for Health Policy Research, AskCHIS https://healthpolicy.ucla.edu/our-work/california-health-interview-survey-chis/access-chis-data (accessed on 15 December 2022).

## Supplementary Materials

The following supporting information can be downloaded at: https://www.mdpi.com/article/10.3390/vision9020040/s1, Table S1: California Health Interview Survey questions addressing COVID-19 mitigation measures.
