# Supplementary material for: The Association Between Sensory Impairment and Adherence to COVID-19 Prevention Measures in the Adult California Health Interview Survey Population
Source: Vision (Basel). 2025 May 2;9(2):40. doi: 10.3390/vision9020040 (PMC12101245; doi:10.3390/vision9020040)
Supplement: Supplementary file 1 [file vision-09-00040-s001.zip › vision-3536797-supplementary.pdf]

vision

Supplementary Materials

The Association Between Sensory Impairment and Adherence to COVID-19 Prevention Measures in the Adult California Health Interview Survey Population

Catherine T. Cascavita, Ahmad Santina, Ken Kitayama, Fei Yu, Victoria L. Tseng and Anne L. Coleman

Table S1: California Health Interview Survey questions addressing COVID-19 mitigation measures

When the COVID-19 vaccine becomes available for you, would you get it?
Yes
No
I have already received at least one dose of the COVID-19 vaccine
When leaving your home in the past week (last 7 days), how often have you done the following: Wore a face covering of any kind?
Never
Sometimes
Usually
Always
I have already received at least one dose of the COVID-19 vaccine
When leaving your home in the past week (last 7 days), how often have you done the following: Sanitized or washed hands
Never
Sometimes
Usually
Always
When leaving your home in the past week (last 7 days), how often have you done the following: Maintained a distance of at least 6 feet from other people
Never
Sometimes
Usually
Always
In the past 30 days, have you participated in any gatherings with persons not living in your household?
Yes
No
